# Treatment with the Probiotic Product Aviguard^®^ Alleviates Inflammatory Responses during *Campylobacter jejuni*-Induced Acute Enterocolitis in Mice

**DOI:** 10.3390/ijms22136683

**Published:** 2021-06-22

**Authors:** Markus M. Heimesaat, Dennis Weschka, Soraya Mousavi, Stefan Bereswill

**Affiliations:** Institute of Microbiology, Infectious Diseases and Immunology, Gastrointestinal Microbiology Research Group, Charité—Universitätsmedizin Berlin, Corporate Member of Freie Universität Berlin, Humboldt-Universität zu Berlin, and Berlin Institute of Health, D-12203 Berlin, Germany; dennis.weschka@charite.de (D.W.); stefan.bereswill@charite.de (S.B.)

**Keywords:** competitive exclusion product, Aviguard^®^, gut microbiota, enteropathogenic infection, *Campylobacter jejuni*, immune-modulatory effects, microbiota-depleted IL-10^−/−^ mice, acute campylobacteriosis model, host–pathogen interaction, probiotic formulations

## Abstract

Prevalences of *Campylobacter (C.) jejuni* infections are progressively rising globally. Given that probiotic feed additives, such as the commercial product Aviguard^®^, have been shown to be effective in reducing enteropathogens, such as *Salmonella*, in vertebrates, including livestock, we assessed potential anti-pathogenic and immune-modulatory properties of Aviguard^®^ during acute *C. jejuni*-induced murine enterocolitis. Therefore, microbiota-depleted IL-10^−/−^ mice were infected with *C. jejuni* strain 81-176 by gavage and orally treated with Aviguard^®^ or placebo from day 2 to 4 post-infection. The applied probiotic bacteria could be rescued from the intestinal tract of treated mice, but with lower obligate anaerobic bacterial counts in *C. jejuni*-infected as compared to non-infected mice. Whereas comparable gastrointestinal pathogen loads could be detected in both groups until day 6 post-infection, Aviguard^®^ treatment resulted in improved clinical outcome and attenuated apoptotic cell responses in infected large intestines during acute campylobacteriosis. Furthermore, less distinct pro-inflammatory immune responses could be observed not only in the intestinal tract, but also in extra-intestinal compartments on day 6 post-infection. In conclusion, we show here for the first time that Aviguard^®^ exerts potent disease-alleviating effects in acute *C. jejuni*-induced murine enterocolitis and might be a promising probiotic treatment option for severe campylobacteriosis in humans.

## 1. Introduction

Foodborne morbidities upon infection with enteropathogens, such as *Salmonella* or *Campylobacter*, cause significant health care-associated and socioeconomic burdens [1]. Particularly, human campylobacteriosis cases caused by *Campylobacter (C.) jejuni* are progressively emerging worldwide, whereas conversely, incidences of *Salmonella* infections are declining [2]. After an incubation period of two to five days, *C. jejuni*-infected patients typically present with symptoms, such as abdominal pain, vomiting, headache, fever, diarrhea, and bloody stools, depending on the virulence of the acquired pathogenic strain and the immune competence of the infected host [3,4]. In most instances, only symptomatic treatment consisting of fluid replacement and application of analgetics is indicated, whereas antibiotics might be required in invasive cases and in subjects at risk for a severe disease course, such as immune-compromised patients [4,5]. Whereas campylobacteriosis is usually resolved within 10 to 14 days weeks post-infection (p.i.), post-infectious autoimmune diseases may occur in rare instances affecting the intestinal tract (i.e., irritable bowel syndrome, celiac disease, chronic inflammatory bowel syndrome), the joints (i.e., reactive arthritis), or even the central nervous system (i.e., Guillain Barré and Miller Fisher syndromes, Bickerstaff encephalitis) [4,6,7,8,9,10]. Notably, both the severity of campylobacteriosis and the risk of the post-infectious collateral damages are highly associated with the sialylation status of the lipooligosaccharide (LOS) derived from the *C. jejuni* cell wall [11]. 

In the intestinal tract of avian vertebrates, *Campylobacter* can be found as commensal residents and usually do not cause clinical signs [12]. The slaughter process of livestock, such as chicken and poultry, harboring *C. jejuni*, however, constitutes the initial step of bacterial entry into the food chain [12,13]. Humans become infected upon ingestion of *C. jejuni*-contaminated undercooked meat products, milk, or surface waters [4,14]. Following successful gastric and duodenal passage, the highly motile Gram-negative bacteria attach to, invade into, and transmigrate through intestinal epithelial cells [15,16]. In the subepithelial tissues, *C. jejuni* bacteria induce recruitment of innate and adaptive immune cells to the site of infection, leading to secretion of pro-inflammatory mediators in the intestinal tract and, in severe and invasive cases, in extra-intestinal and even systemic tissue sites as well [17,18].

In order to lower the incidences of foodborne infections, approaches from “farm to fork” need to be implemented at several positions within the food chain. To reach this goal, competitive exclusion cultures have been developed as early as 1973 [19,20] and proven successful to tackle colonization of *Salmonella* in the intestinal tract of avian livestock [21,22,23]. Among different commercially available competitive exclusion products, Aviguard^®^ constitutes a freeze-dried fermentation product with a longer shelf life compared to others and contains a combination of defined viable commensal bacteria [24]. Recent studies revealed potent competitive exclusion of *Salmonella* and *Clostridium perfringens* from the chicken gut following oral Aviguard^®^ application [24,25]. Experimental data regarding the effects of this commercial competitive exclusion product upon *C. jejuni* colonization and infection of the vertebrate host are scarce, however. This prompted us to test the anti-pathogenic and immune-modulatory effects of oral Aviguard^®^ application in acute *C. jejuni* infection in the frame of the present study. To accomplish this, we used a well-established murine *C. jejuni*-induced infection and inflammation model applying microbiota-depleted interleukin (IL)-10^−/−^ mice. Within a week following oral *C. jejuni* infection, these mice develop acute enterocolitis with extra-intestinal and even systemic pro-inflammatory sequelae due to immune responses that are induced by *C. jejuni* LOS and mediated by Toll-like recptor-4 (TLR-4) signaling [17,26]. The pathogen-induced immunopathology thus resembles important features of acute LOS-driven campylobacteriosis in humans [17,27]. The reliability of the here applied mouse model has been further proven by our recent preclinical intervention studies testing the anti-pathogenic and immune-modulatory properties of distinct molecules, such as vitamin C [28], vitamin D [29], carvacrol [30], urolithin A [31], and resveratrol [32]; of essential oils derived from cardamom [33] and clove [34]; neuropeptides, such as PACAP [35] and NAP [36]; probiotic compounds, such as VSL#3 [37]; and fecal microbiota transplantation [38,39].

## 2. Results

### 2.1. Aviguard^®^ Treatment and Gastrointestinal C. jejuni Infection of Microbiota-Depleted IL-10^−/−^ Mice

**Figure 1:**
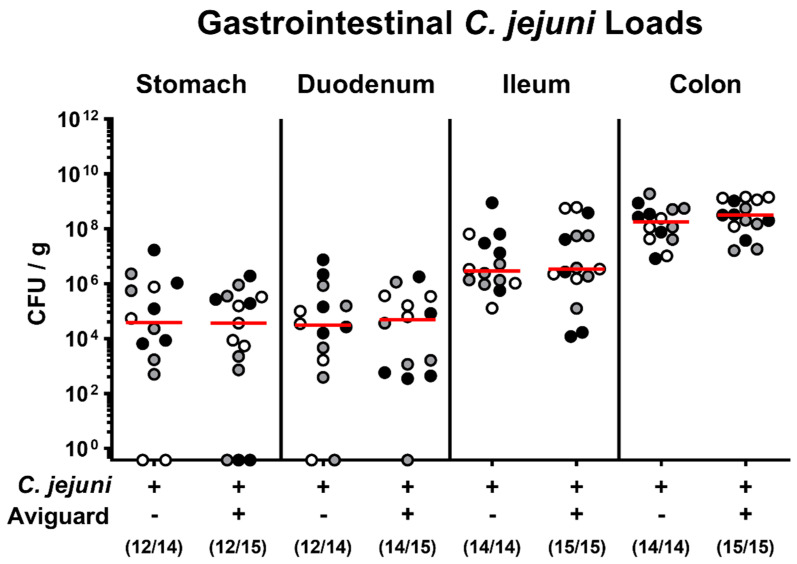
Aviguard^®^ treatment and gastrointestinal *C. jejuni* infection of microbiota-depleted IL-10^−/−^ mice. On days 0 and 1, microbiota-depleted IL-10^−/−^ mice were infected with *C. jejuni* strain 81-176 by gavage and perorally treated with Aviguard^®^ or placebo on days 2, 3, and 4. Upon necropsy (i.e., day 6 post-infection), the gastrointestinal pathogen loads were determined by culture (expressed as colony forming units per g luminal content, CFU/g). Medians (red bars) and numbers of culture-positive mice out of the total number of analyzed animals (in parentheses) are given. Data were pooled from three independent experiments (as indicated by black, white, and gray circles).

Microbiota-depleted IL-10^−/−^ mice were infected with *C. jejuni* strain 81-176 on days 0 and 1 by gavage and perorally subjected to either Aviguard^®^ or placebo treatment on days 2, 3, and 4 p.i. Our cultural analyses revealed that *C. jejuni* stably colonized the intestines of mice from either treatment group with comparably high fecal loads between days 3 and 6 (Supplementary Materials Figure S1). Upon necropsy, the *C. jejuni* cell numbers enumerated in distinct luminal parts of the gastrointestinal tract, namely, in the stomach, duodenum, ileum, and colon, were comparable in respective treatment groups (not significant (n.s.); Figure 1). Hence, oral Aviguard^®^ application did not affect gastrointestinal *C. jejuni* colonization properties. 

### 2.2. Aviguard^®^ Treatment and Intestinal Microbiota Composition of C. jejuni-Infected Microbiota-Depleted IL-10^−/−^ Mice

**Figure 2:**
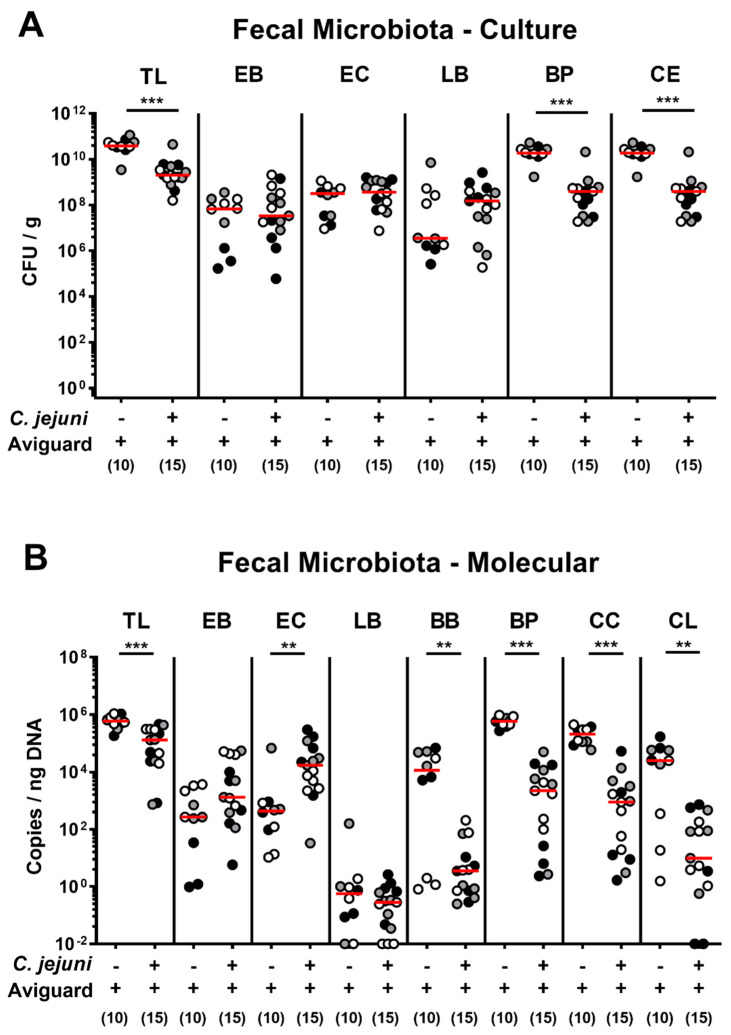
Aviguard^®^ treatment and intestinal microbiota composition of *C. jejuni*-infected microbiota-depleted IL-10^−/−^ mice. Following *C. jejuni* strain 81-176 infection on days 0 and 1, microbiota-depleted IL-10^−/−^ mice were perorally treated with Aviguard^®^ on days 2, 3, and 4 (*n* = 15). The fecal microbiota composition was surveyed on day 6 post-infection applying (**A**) culture (in colony forming units per g, CFU/g) and (**B**) culture-independent (molecular) methods (in copies/ng DNA). Aviguard^®^-treated uninfected animals were included as controls (*n* = 10). Medians (red bars) and significance levels (*p* values) determined by the Mann Whitney *U*-test are shown. Data were pooled from three independent experiments (as indicated by black, white, and gray circles). TL, total bacterial load; EB, enterobacteria; EC, enterococci; LB, lactobacilli; BP, *Bacteroides/Prevotella* species; CE, *Clostridium/Eubacterium* species; BB, bifidobacteria; CC, *Clostridium coccoides* group; CL, *Clostridium leptum* group. ** *p* < 0.01; *** *p* < 0.001.

We further assessed the fecal microbiota composition on day 6 p.i. (i.e., 48 h after the latest of three consecutive oral Aviguard^®^ challenges). Both culture and culture-independent analyses revealed lower total bacterial loads in feces samples following Aviguard^®^ treatment of *C. jejuni*-infected as compared to non-infected counterparts (*p* < 0.001; Figure 2), which was also the case when analyzing obligate anaerobic bacteria, such as bifidobacteria (*p* < 0.01), *Bacteroides/Prevotella* species (*p* < 0.001), and *Clostridium/Eubacterium* species (*p* < 0.001), including *Clostridium coccoides* (*p* < 0.001) and *Clostridium leptum* (*p* < 0.01) groups (Figure 2). Hence, the probiotic bacteria from the Aviguard^®^ suspension could be rescued from the intestinal tract of treated mice, but with lower obligate anaerobic bacterial counts in *C. jejuni*-infected as compared to non-infected mice. 

### 2.3. Aviguard^®^ Treatment and Clinical Outcome in C. jejuni-Infected Microbiota-Depleted IL-10^−/−^ Mice

**Figure 3:**
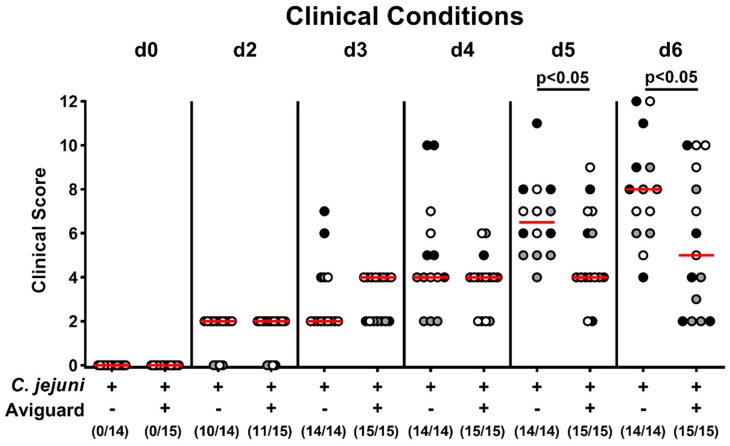
Aviguard^®^ treatment and clinical outcome in *C. jejuni*-infected microbiota-depleted IL-10^−/−^ mice. Following *C. jejuni* strain 81-176 infection on day (d) 0 and d1, microbiota-depleted IL-10^−/−^ mice were perorally treated with Aviguard^®^ or placebo on d2, d3, and d4. Prior and at defined time points post either infection, the clinical outcome was quantified in each mouse by a standardized clinical scoring system. Median (red bars), significance levels (*p* values) determined by the Mann Whitney *U* test, and numbers of clinical score-positive mice out of the total number of analyzed animals (in parentheses) are given. Data were pooled from three independent experiments (as indicated by black, white, and gray circles).

We further surveyed the clinical outcome of *C. jejuni*-infected mice from the Aviguard^®^ and placebo cohorts and quantified induced clinical signs by using a clinical scoring system [40]. As early as day 5 p.i., Aviguard^®^-treated mice were clinically less severely compromised from *C. jejuni*-induced disease as compared to placebo control animals, which also held true for the end of the observation period (*p* < 0.05; Figure 3). Hence, Aviguard^®^ treatment resulted in an improved clinical outcome during acute murine campylobacteriosis.

### 2.4. Aviguard^®^ Treatment and Microscopic Inflammatory Changes in the Colon of C. jejuni-Infected Microbiota-Depleted IL-10^−/−^ Mice

**Figure 4:**
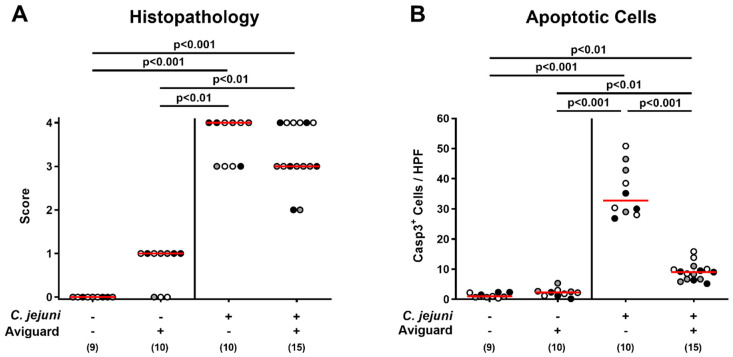
Aviguard^®^ treatment and microscopic inflammatory changes in the colon of *C. jejuni*-infected microbiota-depleted IL-10^−/−^ mice. Following *C. jejuni* strain 81-176 infection on days 0 and 1, microbiota-depleted IL-10^−/−^ mice were perorally treated with Aviguard^®^ or placebo on days 2, 3, and 4. On day 6 post-infection, (**A**) the histopathological changes in the colonic mucosa and lamina propria were quantified by a standardized histopathological scoring system. Furthermore, (**B**) the average numbers of colonic epithelial apoptotic (Casp3^+^) cells were determined microscopically from six high power fields (HPF, 400× magnification) per mouse in immunohistochemically stained colonic paraffin sections. Medians (red bars), significance levels (*p* values) calculated by the Kruskal–Wallis test and Dunn’s post-correction (**A**) or by the ANOVA test with Tukey post-correction (**B**) and numbers of analyzed mice (in parentheses) are indicated. Data were pooled from three independent experiments (as indicated by black, white, and gray circles).

We next quantitatively assessed pathogen-induced histopathological changes in the large intestinal tract in Aviguard^®^-treated mice and controls. On day 6 p.i., mice from either treatment group exhibited markedly increased but comparable histopathological scores indicative of acute enterocolitis (*p* < 0.001 versus naive control mice; Figure 4A). Since apoptosis is considered a valuable parameter for the grading of intestinal inflammatory diseases [41], we additionally enumerated colonic epithelial cells that were positive for cleaved caspase 3 following in situ immunohistochemical staining of colonic paraffin sections. *C. jejuni* infection was associated with increased numbers of apoptotic colonic epithelial cells (*p* < 0.01–0.001), whereas lower caspase 3^+^ cell numbers could be determined in mice from the Aviguard^®^ as compared to the placebo cohort on day 6 p.i. (*p* < 0.001; Figure 4B). Hence, oral Aviguard^®^ treatment not only improved the clinical outcome of *C. jejuni*-induced disease, but also attenuated apoptotic cell responses in the infected large intestines of mice.

### 2.5. Aviguard^®^ Treatment and Pro-Inflammatory Immune Responses in the Intestinal Tract of C. jejuni-Infected Microbiota-Depleted IL-10^−/−^ Mice

**Figure 5:**
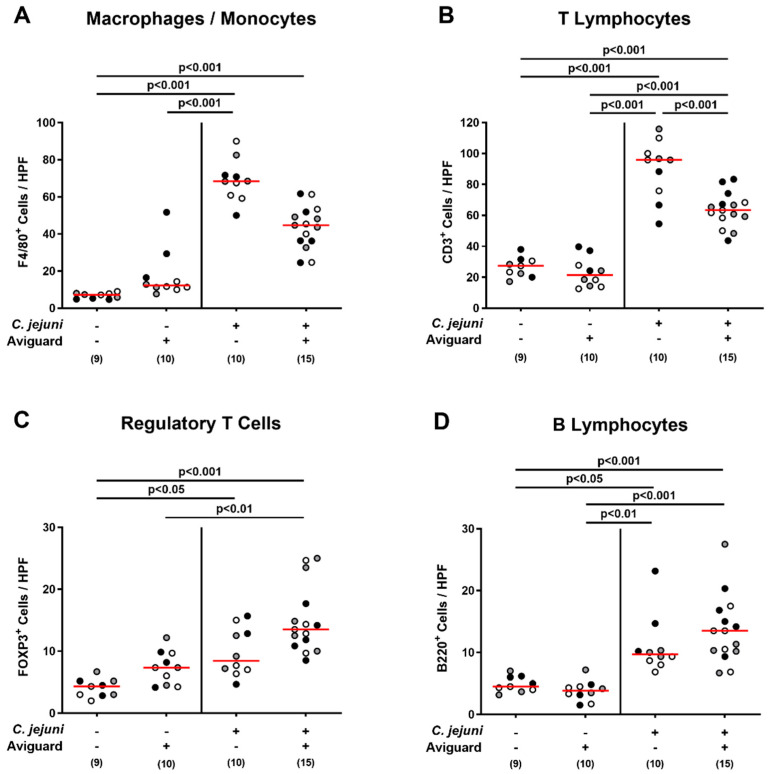
Aviguard^®^ treatment and colonic immune cell responses in *C. jejuni*-infected microbiota-depleted IL-10^−/−^ mice. Following *C. jejuni* strain 81-176 infection on days 0 and 1, microbiota-depleted IL-10^−/−^ mice were perorally treated with Aviguard^®^ or placebo on days 2, 3, and 4. On day 6 post-infection, the average numbers of (**A**) F4/80^+^ cells (macrophages and monocytes), (**B**) CD3^+^ cells (T lymphocytes), (**C**) FOXP3^+^ cells (regulatory T cells), and (**D**) B220^+^ cells (B lymphocytes) were determined microscopically from six high power fields (HPF, 400 × magnification) per mouse in immunohistochemically stained colonic paraffin sections. Medians (red bars), significance levels (*p* values) calculated by the Kruskal–Wallis test and Dunn’s post-correction (**A**,**C**,**D**) or by the ANOVA test with Tukey post-correction (**B**) and numbers of analyzed mice (in parentheses) are indicated. Data were pooled from three independent experiments (as indicated by black, white, and gray circles).

We further determined *C. jejuni*-induced immune cell responses in the large intestines of Aviguard^®^-treated mice, again applying in situ immunohistochemistry. Irrespective of the treatment group, increased numbers of innate immune cells, including F4/80^+^ macrophages and monocytes, were observed in the large intestinal mucosa and lamina propria on day 6 p.i. (*p* < 0.001 versus naive controls; Figure 5A), which also held true for adaptive immune cell subsets, such as CD3^+^ T lymphocytes, FOXP3^+^ regulatory T cells, and B220^+^ B lymphocytes (*p* < 0.05–0.001; Figure 5B–D). The *C. jejuni*-induced increases in colonic T lymphocytes, however, were less pronounced upon Aviguard^®^ as compared to placebo treatment (*p* < 0.001; Figure 5B), whereas a trend towards lower numbers of macrophages and monocytes could be detected in the former versus the latter cohort on day 6 p.i. (n.s.; Figure 5A). 

**Figure 6:**
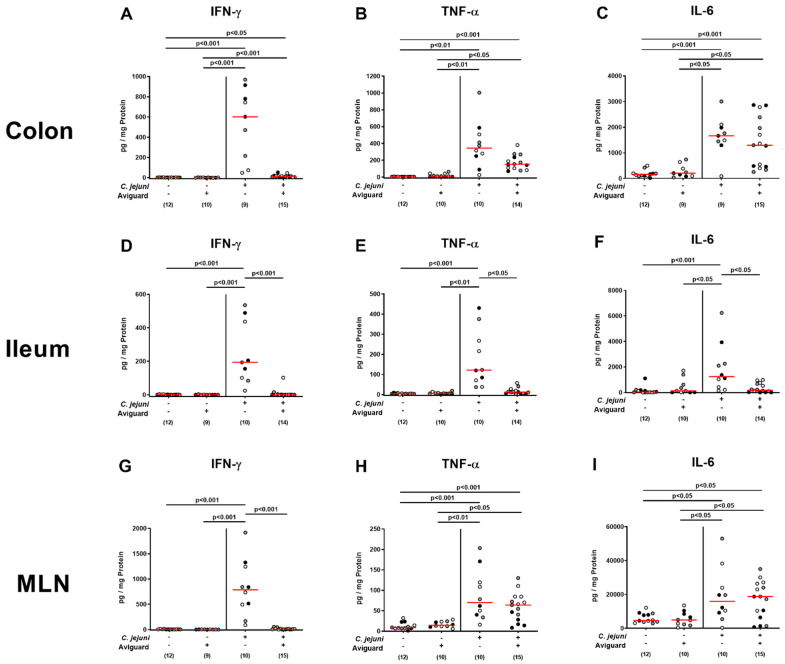
Aviguard^®^ treatment and pro-inflammatory cytokine secretion in the intestinal tract of *C. jejuni*-infected microbiota-depleted IL-10^−/−^ mice. Following *C. jejuni* strain 81-176 infection on days 0 and 1, microbiota-depleted IL-10^−/−^ mice were perorally treated with Aviguard^®^ or placebo on days 2, 3, and 4. On day 6 post-infection, (**A**,**D**,**G**) IFN-γ, (**B**,**E**,**H**) TNF-α, and (**C**,**F**,**I**) IL-6 concentrations were measured in ex vivo biopsies derived from the (**A**–**C**) colon, (**D**–**F**) ileum, and (**G**–**I**) mesenteric lymph nodes (MLNs). Medians (red bars), significance levels (*p* values) calculated by the Kruskal–Wallis test and Dunn’s post-correction, and numbers of analyzed mice (in parentheses) are indicated. Data were pooled from three independent experiments (as indicated by black, white, and gray circles). Definite outliers were removed after identification using the Grubb’s test (α = 0.001).

When assessing intestinal pro-inflammatory cytokine secretion, mice displayed increased interferon-γ (IFN-γ), tumor necrosis factor-α (TNF-α), and interleukin-6 (IL-6) concentrations in colonic ex vivo biopsies within 6 days p.i. (*p* < 0.05–0.001; Figure 6A–C), but with a trend towards lower IFN-γ and TNF-α levels upon Avigurd^®^ as compared to placebo treatment (n.s. due to high standard deviations; Figure 6A,B). Interestingly, in the terminal ileum, increased pro-inflammatory cytokines could be measured in *C. jejuni*-infected mice from the placebo (*p* < 0.001; Figure 6D–F) but not the Aviguard^®^ cohort (n.s. versus naive controls; *p* < 0.05–0.001 versus placebo; Figure 6D–F). This was also the case when assessing IFN-γ concentrations measured in mesenteric lymph nodes (MLN) draining the infected intestinal tract (*p* < 0.001; Figure 6G), whereas TNF-α and IL-6 secretions were comparably increased on day 6 p.i. irrespective of the treatment regimen (*p* < 0.05–0.001; Figure 6H,I). Hence, Aviguard^®^ treatment dampened *C. jejuni*-induced pro-inflammatory immune responses in distinct parts of the intestinal tract. 

### 2.6. Aviguard^®^ Treatment and Pro-Inflammatory Cytokine Secretion in Extra-Intestinal and Systemic Tissue Sites of C. jejuni-Infected Microbiota-Depleted IL-10^−/−^ Mice

**Figure 7:**
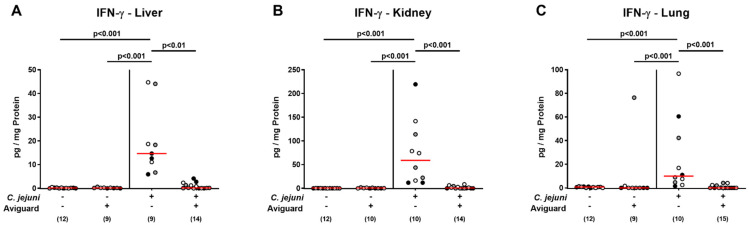
Aviguard^®^ treatment and extra-intestinal IFN-γ secretion in *C. jejuni*-infected microbiota-depleted IL-10^−/−^ mice. Following *C. jejuni* strain 81-176 infection on days 0 and 1, microbiota-depleted IL-10^−/−^ mice were perorally treated with Aviguard^®^ or placebo on days 2, 3, and 4. On day 6 post-infection, IFN-γ concentrations were measured in ex vivo biopsies derived from the (**A**) liver, (**B**) kidneys, and (**C**) lungs. Medians (red bars), significance levels (*p* values) calculated by the Kruskal–Wallis test and Dunn’s post-correction, and numbers of analyzed mice (in parentheses) are indicated. Data were pooled from three independent experiments (as indicated by black, white, and gray circles).

We further addressed whether Aviguard^®^ treatment might also impact pathogen-induced pro-inflammatory cytokine responses in extra-intestinal compartments. In fact, increased IFN-γ concentrations were measured in ex vivo biopsies taken from the liver, kidneys, and the lungs of placebo-treated mice on day 6 p.i. (*p* < 0.001), whereas in the Aviguard^®^ cohort, respective extra-intestinal IFN-γ concentrations did not differ from those assessed in uninfected mice (*p* < 0.001 versus placebo; Figure 7). 

**Figure 8:**
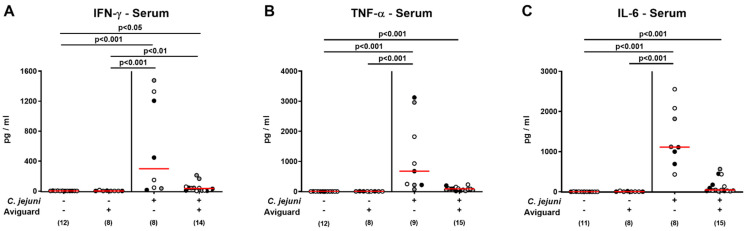
Aviguard^®^ treatment and systemic pro-inflammatory cytokine secretion in *C. jejuni*-infected microbiota-depleted IL-10^−/−^ mice. Following *C. jejuni* strain 81-176 infection on days 0 and 1, microbiota-depleted IL-10^−/−^ mice were perorally treated with Aviguard^®^ or placebo on days 2, 3, and 4. On day 6 post-infection, (**A**) IFN-γ, (**B**) TNF-α, and (**C**) IL-6 concentrations were measured in serum samples. Medians (red bars), significance levels (*p* values) calculated by the Kruskal–Wallis test and Dunn’s post-correction, and numbers of analyzed mice (in parentheses) are indicated. Data were pooled from three independent experiments (as indicated by black, white, and gray circles).

Furthermore, a trend towards lower IFN-γ, TNF-α, and IL-6 concentrations could be observed in serum samples derived from Aviguard^®^ as compared to placebo-treated mice on day 6 p.i. but did not reach statistical significance due to high standard deviations, particularly in the placebo cohort (n.s.; Figure 8). Hence, Aviguard^®^ treatment prevented *C. jejuni*-induced IFN-γ secretion in the extra-intestinal compartments of infected microbiota-depleted IL-10^−/−^ mice. 

## 3. Discussion

In our here presented preclinical intervention trial, we investigated potential pathogen-lowering, disease-alleviating, and immune-modulatory effects upon peroral treatment of microbiota-depleted IL-10^−/−^ mice suffering from acute *C. jejuni*-induced enterocolitis with the commercial competitive exclusion product and probiotic compound Aviguard^®^. On the day of sacrifice (i.e., day 6 p.i.), *C. jejuni* loads did not differ alongside the gastrointestinal tract of verum versus placebo-treated mice. One reason could be that distinct bacterial members of the probiotic product did not fully establish within the intestines. Both our cultural and culture-independent analyses revealed, however, that the high numbers of the main bacterial taxa of the probiotic product could be detected in fecal samples. Nevertheless, we need to emphasize that the *Propionibacterium* and *Fusobacterium* genera were not part of our analytical panel, leaving a gap regarding the insight into the complex probiotic microbiota in the murine host. One might further argue that the time course of the Aviguard^®^ treatment regimen was not sufficient to elicit a biological relevant lowering in intestinal *C. jejuni* numbers. In fact, as early as two days after the latest of three consecutive oral applications of the probiotic suspension on days 2, 3, and 4 p.i., animals were already sacrificed, and the time of treatment could have been too short. Therefore, we are currently performing a longer-term survey of Aviguard^®^-induced effects in wildtype mice that do not succumb to *C. jejuni* infection as opposed to the here applied microbiota-depleted IL-10^−/−^ mice, and are further considering probiotic application via the drinking water starting prior to *C. jejuni* infection applying a prophylactic regimen. Furthermore, competitive exclusion products developed according to the “Nurmi concept” have been shown to be most effective in protecting avian livestock from intestinal *Salmonella* colonization [19,23]. One needs to take into consideration, however, that *Salmonella* and *Campylobacter* species occupy different ecological niches in the intestinal tract [42]. This might explain why competitive exclusion products were not effective against intestinal *C. jejuni* colonization as shown earlier for chicks [43] and why oral Aviguard^®^ application did not affect gastrointestinal *C. jejuni* colonization properties in mice in our present study. 

Given the lack of anti-*Campylobacter* colonization effects, we were even more intrigued by the improved clinical outcome upon Aviguard^®^ treatment during acute murine campylobacteriosis that was paralleled by less pronounced pathogen-induced apoptosis of colonic epithelial cells. In support, various in vitro and in vivo studies demonstrated potent anti-apoptotic properties exerted by distinct probiotic strains and compounds [44]. The probiotic *Lactobacillus rhamnosus* GG strain has been shown to prevent cytokine-induced apoptosis of gut epithelial cells in vitro, for instance [45], whereas less distinct colonic apoptosis was observed in *C. jejuni*-infected mice following treatment with a murine intestinal *Lactobacillus johnsonii* isolate [46] or with the probiotic compound VSL#3 [37].

Moreover, Aviguard^®^ exerted distinct immune-modulatory effects during acute murine campylobacteriosis, affecting both innate and adaptive immune responses upon pathogenic infection. For instance, Aviguard^®^-treated mice displayed approximately one-third lower median numbers of colonic innate immune cell subsets, such as macrophages and monocytes, when compared to placebo counterparts (not significant due to high standard deviations). Furthermore, Aviguard^®^ treatment attenuated *C. jejuni*-induced increases in colonic T lymphocytes, whereas a trend towards approximately 50% higher median numbers of regulatory T cells could be observed in the large intestines of Aviguard^®^ as compared to placebo-treated mice. These results are well in line with previous in vivo studies reporting attenuated increases in colonic innate and adaptive immune cell numbers in *C. jejuni*-infected mice treated with the probiotic compound VSL#3 [37] or with a single probiotic strain (i.e., *Lactobacillus johnsonii*) [46]. 

The potent immune-modulatory effects of oral Aviguard^®^ application to *C. jejuni*-infected mice were further underlined by dampened secretions of pro-inflammatory cytokines, such as IFN-γ, TNF-α, and IL-6, in distinct intestinal compartments, including the colon, ileum, and MLN draining the infected intestines. The anti-inflammatory effects of probiotic strains and compounds have been shown in various in vitro and in vivo studies [44]. For instance, *Lactobacillus acidophilus* downregulated TNF-α expression in intestinal epithelial cells infected with pathogenic enterohemorrhagic *Escherichia coli* (EHEC) cells [47], whereas *Lactobacillus paracasei* decreased *Salmonella Typhi*-induced TNF-α and IL-6 releases in vitro [48].

Furthermore, *Clostridium tyrobutyricum* was shown to suppress colonic TNF-α expression in murine dextran sulfate sodium (DSS)-induced colitis [49]. In support, *L. johnsonii* treatment of *C. jejuni*-infected microbiota-depleted wildtype mice attenuated pathogen-induced intestinal TNF-α secretion [46], which also held true for dampened IFN-γ concentrations measured in the MLN of infected human gut microbiota-associated wildtype mice following oral murine fecal microbiota transplantation (FMT) [50].

Remarkably, the anti-inflammatory effects exerted by oral Aviguard^®^ treatment were not only effective in the intestinal tract of *C. jejuni*-infected mice but could also be observed in extra-intestinal organs as indicated by the IFN-γ concentration measured in the liver, kidneys, and lungs, which returned to the basal range upon probiotic application. Strikingly, probiotic treatment could even alleviate systemic immune responses given that *C. jejuni*-induced increases in TNF-α and IL-6 serum concentrations were assessed in placebo but not in Aviguard^®^ treatment mice, whereas with regards to IFN-γ, at least a trend towards less pronounced systemic secretion could be observed upon treatment of *C. jejuni*-infected mice with the latter versus the former. In support, oral *Lactobacillus johnsonii* application to *C. jejuni*-infected mice was shown to result in attenuated hepatic pro-inflammatory cytokine secretion [46], while murine FMT reversed *C. jejuni*-induced increases in serum TNF-α and IL-6 concentrations measured in infected human microbiota-associated mice [50]. 

One of the key mechanisms underlying *C. jejuni*-induced immune responses is the TLR-4-mediated sensing of LOS derived from the *C. jejuni* cell wall [17]. Previous studies suggest that distinct probiotic bacterial strains exert their anti-inflammatory effects via the downregulation of lipopolysaccharide (LPS)-induced pro-inflammatory pathways [44,51]. For instance, different *Lactobacillus reuteri* strains were shown to decrease secretion of pro-inflammatory cytokines, including TNF-α and IL-6, in newborn rats suffering from necrotizing enterocolitis (NEC), which was accompanied by probiotic TLR-4 signaling inhibition via the nuclear factor-kappa B (NF-kB) pathway [52]. Even though it has not been addressed to date, it is tempting to speculate that the pronounced intestinal, extra-intestinal, and systemic anti-inflammatory effects observed within four days of triple oral Aviguard^®^ applications during acute *C. jejuni*-induced enterocolitis might be due to interference with the LOS-induced and TLR-4 dependent signaling pathway. 

From the microbiological ecological point of view, it is interesting to note that the probiotic bacteria from the Aviguard^®^ suspension could be rescued from the intestinal tract of treated mice, but with lower obligate anaerobic bacterial counts in *C. jejuni*-infected as compared to non-infected mice. In fact, we cannot provide a conclusive explanation for this observation, but we raise the following hypotheses: firstly, there might be direct competition for nutrients and niches between the probiotic bacterial species and the pathogen, resulting in deprived anaerobic bacterial counts. Secondly, pathogen-induced changes within the intra-luminal milieu could result in a predominance of molecules, including oxygen, exerting toxic effects to obligate anaerobic bacteria. Thirdly, the interplay between enteropathogens, such as *C. jejuni*, and commensal gut bacteria, such as anaerobic species, might result in interferences in energy, including H_2_ metabolism, leading to ecological shifts within the intestinal lumen [53]. Notably, anaerobic subcultures from cecal luminal samples did not protect chicks from *Campylobacter* colonization, however [43].

In conclusion, we provide evidence for the first time that the probiotic compound Aviguard^®^ exhibits potent immune-modulatory and in turn, disease-alleviating but not anti-pathogenic effects during acute *C. jejuni*-induced murine enterocolitis and constitutes a promising adjunct treatment option for acute campylobacteriosis in humans. 

## 4. Material and Methods

### 4.1. Microbiota-Depleted IL-10^−/−^ Mice

Conventional IL-10^−/−^ mice (C57BL/6j background) were reared in the Forschungsinstitute für Experimentelle Medizin, Charité—University Medicine Berlin (Berlin, Germany), housed in cages equipped with filter tops under standard conditions (22–24 °C room temperature, 55 ± 15% humidity, 12 h light/12 h dark cycle) in an experimental semi-barrier facility and had free access to autoclaved standard food pellets (ssniff R/M-H, V1534-300, Sniff, Soest, Germany) and tap water. Female and male 3-week-old litter-mate mice were treated with broad-spectrum antibiotics, in order to deplete the commensal gut microbiota as reported earlier [41,54]. In brief, mice were transferred to sterile cages (maximum of 4 animals per cage) and treated for eight weeks with an antibiotic cocktail consisting of ampicillin plus sulbactam (1 g/L; Dr. Friedrich Eberth Arzneimittel, Ursensollen, Germany), imipenem (250 mg/L; Fresenius Kabi, Bad Homburg, Germany), vancomycin (500 mg/L; Hikma Pharmaceuticals, London, UK), metronidazole (1 g/L; B. Braun, Melsungen, Germany), and ciprofloxacin (200 mg/L; Fresenius Kabi, Bad Homburg, Germany) added to autoclaved drinking water (ad libitum). Microbiota-depleted mice were continuously kept and handled under strict aseptic conditions. Three days before infection, the antibiotic treatment was withdrawn, and mice received autoclaved tap water instead. 

### 4.2. Campylobacter jejuni Infection

From a frozen *C. jejuni* strain 81-176 stock, bacteria were thawed and harvested on karmali agar plates and on columbia agar including 5% sheep blood (both from Oxoid, Wesel, Germany). Age- and sex-matched microbiota-depleted IL-10^−/−^ mice (4 months of age) were infected perorally with 10^9^ colony forming units (CFU) of the pathogen on days 0 and 1 by gavage (in 0.3 mL sterile phosphate-buffered saline (PBS; Gibco, Life Technologies, Loughborough, UK)).

### 4.3. Treatment of Mice with Commercial Exclusion Product Aviguard^®^

Approximately half an hour before oral application, 1 g of the commercial exclusion product Aviguard^®^ (from Lallemand Animal Nutrition, Worcestershire, UK) was dissolved in 10 mL phosphate-buffered saline (PBS; Gibco, Life Technologies, Loughborough, UK). On days 2, 3, and 4 p.i., mice received 0.3 mL of the bacterial suspension via the peroral route (by gavage). The compound contains the following bacterial species (approximately 10^9^ CFU per g, as indicated by the company): *Escherichia coli*, *Citrobacter* species, *Enterococcus* species (*E. faecalis*, *E. faecium*), *Lactobacillus* species (*L. casei*, *L. plantarum*), *Bacteroides* species, *Clostridium* species (*C. sporogenes*), *Eubacterium* species, *Propionibacterium* species, *Fusobacterium* species, and *Ruminococcus* species [55].

### 4.4. Gastrointestinal Colonization by C. jejuni

For quantification of gastrointestinal pathogen loads, feces and luminal samples from the stomach, duodenum, ileum, and colon were homogenized with a sterile pestle. Then, serial dilutions were streaked onto karmali and columbia (with 5% sheep blood) agar plates (both from Oxoid, Wesel, Germany) and incubated in a jar containing CampyGen gas packs (from Oxoid, Wesel, Germany) under microaerophilic conditions (for at least 48 h, 37 °C).

### 4.5. Cultural Intestinal Microbiota Analysis

The microbiota compositions of fecal and gastrointestinal luminal samples were performed by culture. Therefore, respective samples were homogenized in sterile PBS (Gibco, Life Technologies, Loughborough, UK) and serial dilution plated onto distinct solid media and grown at 37 °C for at least two days under aerobic conditions (in a CO_2_ chamber) and under anaerobic conditions (in a jar containing an AnaeroGen Gas Pack, Oxoid, Wesel, Germany) as reported elsewhere [54,56,57]. In brief, for quantitative assessment of total bacterial loads, bacterial colonies were enumerated on columbia agar supplemented with 5% sheep blood (incubated under aerobic and anaerobic conditions). For quantification of enterobacteria and enterococci, respective bacterial colonies were counted on McConkey agar plates (Oxoid, Wesel, Germany) and on columbia CNA agar plates supplemented with 5% sheep blood, colistin, and nalidixic acid (BD Biosciences, Heidelberg, Germany), respectively, following aerobic incubation. Lactobacilli, *Clostridium/Eubacterium* species, and *Bacteroides/Prevotella* species were grown on MRS (Rogosa) agar (Oxoid, Wesel, Germany), on columbia agar supplemented with 5% sheep blood (Oxoid, Wesel, Germany), and on Schaedler-KV agar (supplemented with 5% sheep blood, kanamycin and vancomycin; BD Biosciences, Heidelberg, Germany), respectively, all upon anaerobic incubation.

### 4.6. Molecular Intestinal Microbiota Analysis

For the survey of fastidious and uncultivable bacteria, we additionally performed culture-independent molecular (i.e., 16S rRNA based) analyses of the bacterial suspensions and fecal samples by quantitative real-time polymerase chain reaction (qRT-PCR) as described in detail earlier [50,57,58]. In brief, the total genomic DNA was extracted from respective samples, quantitated by using Quant-iT PicoGreen reagent (Invitrogen, UK), and adjusted to 1 ng per µL. Then, total eubacterial loads as well as the main intestinal bacterial groups, including Enterobacteriaceae, Enterococcus species, Lactobacillus species, Bifidobacterium species, *Bacteroides/Prevotella* species, *Clostridium leptum* group, and *Clostridium coccoides* group, were assessed with species-, genera-, or group-specific 16S rRNA gene primers (Tib MolBiol, Berlin, Germany) and numbers of 16S rRNA gene copies per ng DNA of each sample were determined.

### 4.7. Clinical Outcome

Prior and at defined time points post Aviguard^®^ challenge, we quantitatively surveyed the clinical signs in *C. jejuni*-infected mice applying defined clinical scores (maximum 12 points), addressing the clinical aspect/wasting (0: normal; 1: ruffled fur; 2: less locomotion; 3: isolation; 4: severely compromised locomotion, pre-final aspect), the abundance of blood in feces (0: no blood; 2: microscopic detection of blood by the Guajac method using Haemoccult, Beckman Coulter/PCD, Krefeld, Germany; 4: macroscopic blood visible), and stool consistency (0: formed feces; 2: pasty feces; 4: liquid feces) as reported earlier [40].

### 4.8. Sampling Procedures

Mice were sacrificed by CO_2_ asphyxiation on day 6 p.i. Cardiac blood (for serum cytokine measurements), gastrointestinal luminal samples, and ex vivo biopsies from distinct intestinal tissue sites, such as colon, ileum, and MLN, and from extra-intestinal organs, including lungs, kidneys, and liver, were collected under aseptic conditions. Large intestinal samples were taken from each mouse in parallel for microbiological and immunohistopathological and immunological analyses.

### 4.9. Histopathology

Microscopic inflammatory changes were quantitated in colonic explants upon immediate fixation in 5% formalin, paraffin embedding, and staining of sections (5 μm) with hematoxylin and eosin (H&E) by using standardized histopathological scores as reported earlier [59]: Score 1: minimal inflammatory cell infiltrates in the mucosa with intact epithelium. Score 2: mild inflammatory cell infiltrates in the mucosa and submucosa with mild hyperplasia and mild goblet cell loss. Score 3: moderate inflammatory cell infiltrates in the mucosa with moderate goblet cell loss. Score 4: marked inflammatory cell infiltration into in the mucosa and submucosa with marked goblet cell loss, multiple crypt abscesses and crypt loss.

### 4.10. In Situ Immunohistochemistry

Quantitative in situ immunohistochemical analyses were performed in immediately fixed 5-µm-thin colonic paraffin sections as recently reported [60,61]. In brief, in order to detect apoptotic epithelial cells, macrophages and monocytes, T lymphocytes, regulatory T cells, and B lymphocytes, 5-µm-thin colonic paraffin sections were stained with primary antibodies directed against cleaved caspase 3 (Asp175, Cell Signaling, Beverly, MA, USA, 1:200), F4/80 (no. 14-4801, clone BM8, eBioscience, San Diego, CA, USA; 1:50), CD3 (no. N1580, Dako, Glostrup, Denmark; 1:10), FOXP3 (clone FJK-165, no. 14-5773, eBioscience, San Diego, CA, USA; 1:100) and B220 (no. 14-0452-81, eBioscience San Diego, CA, USA; 1:200), respectively. An independent investigator enumerated positively stained cells by applying light microscopy and assessed the mean number of positive cells in each sample within six high power fields (HPF, 0.287 mm^2^, 400× magnification).

### 4.11. Pro-Inflammatory Cytokine Measurements

Ex vivo biopsies obtained from the colon and ileum (longitudinally cut strips of approximately 1 cm^2^) as well as from MLN (3 lymph nodes) were washed in sterile PBS (Gibco, Life Technologies, Loughborough, UK) and transferred to 24-flat-bottom well culture plates (Nunc, Darmstadt, Germany) containing 500 μL serum-free RPMI 1640 medium (Gibco, Life Technologies, Loughborough, UK) plus penicillin (100 µg/mL) and streptomycin (100 µg/mL; Biochrom, Berlin, Germany). After incubation at 37 °C for 18 h, respective culture supernatants and serum samples were tested for IFN-γ, TNF-α, and IL-6 by the Mouse Inflammation Cytometric Bead Assay (CBA; BD Biosciences, Heidelberg, Germany) in a BD FACSCanto II flow cytometer (BD Biosciences, Heidelberg, Germany).

### 4.12. Statistical Analyses

Medians and significance levels were calculated using GraphPad Prism (version 8, San Diego, CA, USA). Normalization of data sets was assessed by the Anderson–Darlin test. Not normally distributed data were pairwise compared by the Mann–Whitney test. For multiple comparisons, the Kruskal–Wallis test with Dunn’s post-correction was used for not normally distributed data, whereas the one-sided ANOVA with Tukey post-correction was applied for normally distributed data. Two-sided probability (*p*) values ≤ 0.05 were considered significant. Definite outliers were removed after identification with the Grubb’s test (α = 0.001). Data were pooled from three independent experiments.

## Data Availability

Available from corresponding Author.

## Supplementary Materials

The following are available online at https://www.mdpi.com/article/10.3390/ijms22136683/s1. Figure S1. Fecal pathogen loads over time following oral Aviguard^®^ versus placebo application to *C. jejuni*-infected microbiota-depleted IL-10^−/−^ mice.
