# Supplementary material for: Treatment with the Probiotic Product Aviguard® Alleviates Inflammatory Responses during Campylobacter jejuni-Induced Acute Enterocolitis in Mice
Source: Int J Mol Sci. 2021 Jun 22;22(13):6683. doi: 10.3390/ijms22136683 (PMC8269033; doi:10.3390/ijms22136683)
Supplement: Supplementary file 1 [file ijms-22-06683-s001.zip › ijms-1227925-supplementary.pdf]

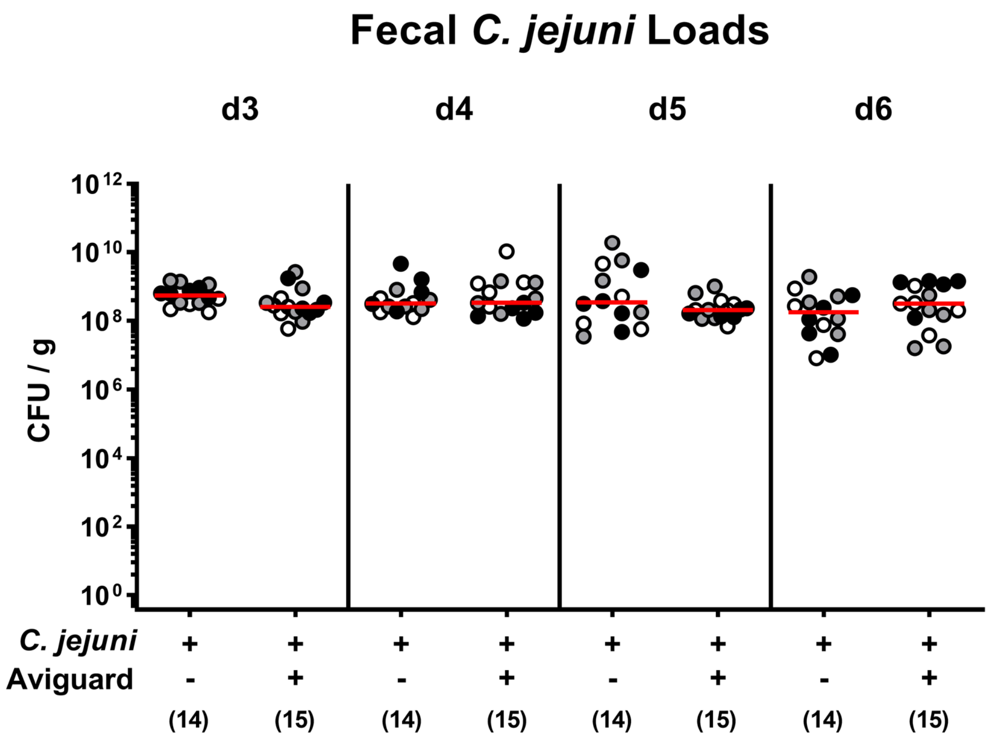

Figure S1. Fecal pathogen loads over time following oral Aviguard® versus placebo application to *C. jejuni*-infected microbiota-depleted IL-10$^{-/-}$ mice.
